# Dimensions of Health Security—A Conceptual Analysis

**DOI:** 10.1002/gch2.201700003

**Published:** 2020-07-28

**Authors:** Preslava Stoeva

**Affiliations:** ^1^ Department of Global Health & Development London School of Hygiene and Tropical Medicine 15‐17 Tavistock Place London WC1H 9SH UK

**Keywords:** conceptual framework, cooperative security, health security, security for individuals

## Abstract

Discussions of the politics and practicalities of confronting health security challenges—from infectious disease outbreaks to antimicrobial resistance and the silent epidemic of noncommunicable diseases—hinge on the conceptualization of health security. There is no consensus among analysts about the specific parameters of health security. This inhibits comparative evaluation and critique, and affects the consistency of advice for policymakers. This article aims to contribute to debates about the meaning and scope of health security by applying Baldwin’s (1997) framework for conceptualizing security with a view to propose an alternative framing. Asking Baldwin’s concept‐defining questions of the health security literature highlights how implicit and explicit assumptions currently place health security squarely within a narrow traditionalist analytical framework. Such framing of health security is inaccurate and constraining, as demonstrated by practice and empirical observations. Alternative approaches to security propose that security politics can also be multiactor, cooperative, and ethical, while being conscious of postcolonial and feminist critique in search of sustainable solutions to existential threats to individuals and communities. A broader conceptualization of health security can transform the politics of health security, improving health outcomes beyond acute crises and contribute to broader security studies’ debates.

## Graphical abstract

This is a critical reflection on the mainstream conceptualization of health security, examined through the lens of Baldwin’s concept of security framework with a view to propose an alternative framing of health security, which draws more effectively on alternative and critical security conceptualizations and practice. The need for such framing is illustrated by recent health emergencies and the COVID‐19 pandemic.

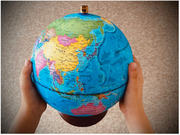

## 1 Introduction

Discussions of health security periodically climb up the global political agenda, mostly in response to global health‐related concerns and challenges—from public health emergencies of international concern and pandemics, e.g., H1N1, Ebola, Zika, and the 2019 COVID‐19 pandemic,^[^ ^1^, ^2^, ^3^, ^4^, ^5^, ^6^, ^7^, ^8^ ^]^ to concerns about antimicrobial resistance, defined by the World Health Organisation (WHO) as a fundamental threat to human health, development and security,^[^ ^9^ ^]^ and the “silent” noncommunicable disease pandemic.^[^ ^10^, ^11^ ^]^ Academic interest in and appetite for health security analysis has not abated either, as indicated by recent “Lancet” contributions.^[^ ^5^, ^12^, ^13^, ^14^, ^15^ ^]^ The response to the global COVID‐19 pandemic is a stark example of health security politics, despite the pandemic not being currently labeled as a health security concern in political discourse. With high levels of morbidity and mortality globally and a highly contagious pathogen this pandemic is a prime and unprecedented example of a global health security threat. Analyses of the politics and practicalities of confronting health‐related threats, of policy options and institutional approaches, however, hinge on the way these challenges are constructed and on the way the dimensions of the concept of health security are charted.

In 2015 Horton and Das noted that there was no simple definition of health security.^[^ ^5^ ^]^ Indeed, there has been little consensus among analysts over the meaning and parameters of health security.^[^ ^16^, ^17^, ^18^, ^19^ ^]^ These disagreements, Aldis argues, have effects beyond analytical debates, as they hinder communications and collaboration on global health initiatives, creating confusion and mistrust among stakeholders.^[^ ^18^ ^]^ They also inhibit comparative evaluation, critique of existing analysis, or the possibility of consistent policy recommendations. Conceptual analyses of the two constitutive parts of health security illustrate the difficulties of coming up with a simple definition and the inherent tensions and contestations in such debates. Given these difficulties, it is argued here that a framework for conceptual analysis of “health security,” instead of a fixed definition, would provide valuable space to evaluate the key features of existing analysis, the explicit and implicit assumptions about the nature and parameters of health security politics underpinning current policy responses, as well as possible alternative conceptualizations and ways of thinking about health security.

Health security politics is a burgeoning and contested field of analysis and practice with the potential to affect security thinking beyond its own parameters. This article aims to contribute to debates both about the scope and meaning of health security and about the scope and meaning of security more broadly. To achieve this, it first presents a brief review of the scope and focus of contemporary conceptual debates of health security; second, it applies Baldwin’s^[^ ^20^ ^]^ framework for the concept of security to demonstrate how conservative the current definition of health security is; and thirdly, it proposes an alternative framing with a view to demonstrate the benefits of thinking about health security in broader more inclusive ways, which has the potential to improve global responses to current and future health‐related challenges.

## 2 Competing Conceptualizations of Health Security

Conceptualizations of health security emerged over time in response to specific health challenges, political and institutional developments.^[^ ^21^, ^22^ ^]^ Academic analysis has broadly (with a few exceptions) sought to fit health into mainstream security studies paradigms instead of using health to broaden security debates through reflection on practice and the engagement of emerging security paradigms. There is a notable reliance on the securitization framework promoted by the Copenhagen School^[^ ^23^ ^]^ to explain the rise to prominence of health concerns in security politics, but that, it will be argued here has supported, validated, even justified a narrow, privileged view of health security.

In his overview of the multiple meanings of health security, McInnes argues that just like “security,” “health security” is “essentially contested” and it is therefore not possible to reach an agreement on the meaning and application of the term.^[^ ^19^ ^]^ He observes that the different framings of health security are “not amenable to a single set of agreed criteria” because they have been constructed to serve a particular purpose, are premised on different sets of assumptions, have different uses and privilege diverse interests and agendas.^[^ ^19^ ^]^ Such observations, however, default on the need for systematic discussion and critical reflection on the way health security has been conceptualized, or the interests that such conceptualization might be serving.

The following brief review of the most common health security conceptualizations in historic context highlights the limited engagement with existing specialist knowledge both from across the spectrum of security studies paradigms including critical security studies, postcolonial and feminist approaches, with specialist knowledge of foreign policy, foreign policy analysis, governance, and global health governance or with key developments in practice. The failure to engage with the broader spectrum of existing knowledge constrains the diversity of discussions, interdisciplinary dialog and learning, as well as the possibility of progressive policy impact.

### 2.1 Health Security as a National Security and a Foreign Policy Issue

Links between national security and infectious disease outbreaks were initially identified by US analysts in the mid to late 1990s.^[^ ^24^, ^25^, ^26^, ^27^, ^28^, ^29^ ^]^ The promotion of health in developing countries was included in US National Security Strategies (NSS) in 1994–1996.^[^ ^30^, ^31^, ^32^ ^]^ Infectious diseases were described as a significant challenge in low and middle‐income countries, contributing to a slowdown in economic growth. The 1999 US “NSS A National Security Strategy for a New Century” was the first such document to state that health problems “can undermine the welfare of US citizens, and compromise our national security, economic and humanitarian interests abroad for generations.”^[^ ^33^ ^]^ Health issues of particular interest to the United States included food‐borne diseases from imported foodstuffs, new and emerging infectious diseases and HIV/AIDS. The Bush administration re‐iterated concerns about the threats posed by biological weapons and pandemic health threats but did not prioritize health‐related security as much.^[^ ^34^ ^]^ Considerations about national and global health made their way back into the US NSS in 2010 and 2015 under the Obama administration. Pandemic disease was considered a threat to “the security of regions and the health and safety of the American people.”^[^ ^35^ ^]^

The first UK National Security Strategy published in 2008 claimed it was premised on a broader conceptualization of national security that included “threats to individual citizens and to our way of life, as well as to the integrity and interests of the state”^[^ ^36^ ^]^ and listed infectious diseases (particularly the threat of a global influenza pandemic) and bioterrorism as national security concerns. The 2010 UK National Security Strategy defined the risk of a severe influenza pandemic as one of the top three civil emergencies risks.^[^ ^37^ ^]^ While the qualification “broader conceptualization” is intended to refer to a move away from concerns of defense and military security, the narrow focus on bioterrorism and communicable disease is symptomatic of traditional, state‐centric thinking about security from acute threats originating outside of it.

Fidler (2003) provides detailed analysis of the practical ways in which the linkages between public health and national security have emerged. He concludes that the realpolitik perspective on national security is driving the development of the concept of public health security in the United States despite three other possible formulations—common, human and ecological security.^[^ ^38^ ^]^ Rushton (2011) also observes that health security continues to be framed in narrow traditional terms as national security and underpinned by particular concerns of interest to rich industrialized states, which shape a narrow discourse that largely disregards the needs of the Global South.^[^ ^39^ ^]^ McInnes adds that “health issues are not identified as national security risks by reference to an explicit set of criteria but rather have arisen in an ad hoc manner and been agreed to intersubjectively by key national and international actors.”^[^ ^19^ ^]^ These observations inadvertently contribute to normalizing dominant political discourses about the paramount nature of the national interest, the centrality of the interests of powerful states and the relevance of only acute health threats to security thinking.

National security is often considered the key objective of foreign policy. HIV/AIDS was framed as a foreign policy problem by the Clinton Administration’s Interagency working group on emerging and re‐emerging infectious diseases’ report “Infectious Disease: A Global Health Threat,” and the National Intelligence Council’s report “The Global Infectious Disease Threat and its Implications for the United States.” Fidler’s analysis, however, mistook US’ foreign policy focus on emerging and re‐emerging communicable diseases and bioterrorism for a global trend and a normative shift, claiming that health had achieved “pre‐eminent political value for 21st century humanity.”^[^ ^40^ ^]^ Kickbusch (2002) argued that the US had shaped the international agenda to fit in with its national interests and priorities and in doing so had preferenced a “unilateral hegemonic approach” to multilateral cooperation.^[^ ^41^ ^]^ The implications of US leadership in shaping the international health security agenda remain understudied, and yet critically relevant to what is included and excluded from that agenda.

The political recognition of health issues as a matter of foreign policy by other states was marked by the 2007 Oslo Ministerial Declaration on global health and foreign policy (Ministers of Foreign Affairs of Brazil, France, Indonesia, Norway, Senegal, South Africa, and Thailand, 2007) and the adoption of UN General Assembly resolution 63/33, which “recognizes the close relationship between foreign policy and global health and their interdependence and… urges member states to consider health issues in the formulation of foreign policy.”^[^ ^42^ ^]^ The reason given for linking health policy with foreign policy at the international level was that health problems of global magnitude were deemed to require cooperative solutions. The international community continues to struggle, however, to find such cooperative state‐led solutions, as illustrated by the response to the Ebola crisis^[^ ^43^ ^]^ and by the current response to the COVID‐19 pandemic, which has broadly been marked by states leading individual responses. Considering the inclusion of health issues on states’ foreign policy agendas as novelty, of course, ignores a long tradition of state cooperation dating back at least to the 19th century.^[^ ^44^ ^]^

The analysis of health concerns as issues of national security and foreign policy suffers from some prominent shortcomings. Discussions of foreign policy and health make virtually no reference to analytical frameworks from the field of foreign policy and foreign policy analysis, failing to draw on its methodologies, paradigms and empirical knowledge. In other words, the presence of health on foreign policy agendas and its construction as a threat to national security is observed in practice, but not sufficiently interrogated in analytical terms. Furthermore, studies often assume generalizability beyond one state (most commonly the USA), which has skewed analysis and aligned it almost exclusively with dominant paradigms of great‐power politics, failing to reflect on how health features in the foreign policy agendas of a broader spectrum of states.

### 2.2 Health as an International Security Concern

Health concerns have been conceptualized as international security challenges in a number of high‐level policy pronouncements. In her role as Director General of the World Health Organisation (WHO), Gro Harlem Brundtland argued that health was an underlying determinant of development, security, and global stability and that in an interdependent world the functional separation between domestic and international health policy lost its meaning.^[^ ^45^ ^]^ Brundtland advocated international cooperation in addressing health‐related global threats because “[a] world where a billion people are deprived, insecure and vulnerable, is an unsafe world.”^[^ ^45^ ^]^ These observations are poignantly relevant 17 years on in the fight against the COVID‐19 pandemic.

Further recognition of health issues as “threats to international peace and security” is evident in UN Security Council resolutions. Security Council Resolution 1308 (2000) acknowledged the growing impact of the HIV/AIDS pandemic in Africa on social instability and emergency situations, and stressed that if left unchecked, it “may pose a risk to stability and security.”^[^ ^46^ ^]^ This historic resolution was followed by two others—Resolution 1983 (2011) and 2177 (2014) respectively on HIV/AIDS and Ebola. The EU Security Strategy (2003) is another example of framing health as an international security threat. It links infectious diseases to poverty, economic failure, political problems and ultimately violent conflict,^[^ ^47^ ^]^ and also notes the threat posed by the potential rapid spread of new diseases and the devastation caused by the HIV/AIDS pandemic.

The concept of international security has been discussed almost in passing in the security literature by Buzan (1991), describing it as focused “on the sources and causes of threats, [with its]… purpose being not to block or offset the threats, but to reduce or eliminate them by political action.”^[^ ^48^ ^]^ This definition is in contrast to his discussion of national security, which focuses on “reducing the vulnerabilities of the state… by increasing self‐reliance, or by building countervailing forces to deal with specific threats.^[^ ^48^ ^]^ The concept of ‘world security” is in the words of Ken Booth “more encompassing than the notion of international security… [including] the structures and processes within human society… that work toward the reduction of the threats and risks that determine individual and group lives.”^[^ ^49^ ^]^ Both concepts—of international and world security—are very relevant to thinking about mechanisms to reduce health insecurity, but are rarely used by analysts to frame interrogations of the nature, scope and focus of health security politics. It is curious that there has been so little conceptual analysis of these political statements pronouncing health as a global/international security concern.

### 2.3 Health Security as Human Security

Health security as an aspect of human security has received the least attention in the health security literature. Health is one of the seven areas identified by UNDP’s “Human Development Report” (1994) as pertinent to human security. Much contemporary analysis, however, focuses on the mechanisms through which health affects human security, and not on the political or policy implications of promoting and pursuing human security, or indeed on what such health security policy might look like. Health as a human security issue is broadly defined and premised on WHO’s definition of health as “a state of complete physical, mental, and social well‐being and not merely the absence of disease or infirmity.”^[^ ^50^ ^]^ This in turn means a broader view of the range of relevant health threats—going beyond communicable diseases and bioterrorism, to include for example noncommunicable diseases, neglected tropical diseases; as well as considerations about the social determinants of health. This view considers “the many other health challenges faced by more vulnerable groups who are amongst those most affected by the burden of disease.”^[^ ^51^ ^]^ Proponents of this approach further note that health threats are experienced most acutely by marginalized groups and communities and that gains in health anywhere in the world benefit everyone everywhere.^[^ ^52^ ^]^ Takemi et al. argue that a human security approach can contribute to improvements in health because it focuses on the needs of communities, recognizes people’s vulnerabilities and strengthens the interface between protection and empowerment.^[^ ^53^ ^]^

Critics have argued that human security does not have sufficient political traction,^[^ ^19^, ^39^ ^]^ building on critiques of the concept as being too broad to serve as a guide for academic research or governmental policy making.^[^ ^54^ ^]^ While the term “human security” might have lost political traction, however, the value of promoting human‐centered security is deeply embedded in existing human rights norms and humanitarian law, as well as doctrines such as the responsibility to protect. This calls for further substantive analysis of the positioning of health security in a broader normative and political context. As the Commission on Human Security has suggested individual and state security need to be considered as complementary—an avenue for analysis that remains largely unexplored.^[^ ^52^ ^]^

All the different framings above share a conscious or an unconscious drive to embed public health concerns into existing frameworks for thinking about security. As Barkawi and Laffey posit, however, “security relations today [sic] are about the contradictions between old security logics and new security problematics.”^[^ ^55^ ^]^ Health security politics provide an accurate illustration of these tensions between understandings of security as a zero‐sum game between great powers and the everyday realities of challenges posed by disease and ill‐health, affecting the life and well‐being many across the world. These tensions, however, cannot be reconciled by the old security theories, premised on old security logics, because they are partly the cause of the problem.

Analysis, instead, needs to draw on practice and emerging security paradigms. New frameworks for analyzing security politics include, but are not limited to cooperative security,^[^ ^56^, ^57^, ^58^, ^59^, ^60^ ^]^ multiactor approaches to security politics,^[^ ^61^, ^62^ ^]^ ethical security studies.^[^ ^63^ ^]^ These are particularly relevant to analyzing health security politics, which, as will be discussed, include complex interactions between public and private actors, aim to address issues that transcend national borders, and affect individuals and groups more acutely than states. A synthesis between alternative security approaches and empirical insight would provide a solid foundation for a more pragmatic understanding of how the political realms of health and security intersect and indeed interact.

## 3 The Concept of Health Security

Conceptual clarity is key in situating analysis, generating comparable findings, and facilitating understanding of commonality and diversity, argues Baldwin.^[^ ^20^ ^]^ It is the first step in facilitating meaningful scholarly engagement and the development of policy proposals that are “comparable with each other and with the policies of pursuing other goals” and can easily be evaluated by end users.^[^ ^20^ ^]^ The questions that define the concept of security are: “Security for whom? Security for which values? How much security? From what threats? By what means? At what cost?”^[^ ^20^ ^]^ This framework is applied here with a view to highlighting implicit and explicit assumptions made about the nature and scope of health security politics in the existing literature.

Some of the questions from Baldwin’s framework have been used to frame discussions of health security already in two influential works by Simon Rushton—“Global Health Security: Security for Whom? Security from What?”^[^ ^39^ ^]^ and Security and Public Health.^[^ ^64^ ^]^ There are two main issues with these works—first, Rushton’s analysis, as will be discussed below, is cautious and does not push conceptual boundaries far enough to explore the outer limits of health security; and secondly, these works only partially engage with Baldwin’s framework, meaning that Rushton’s analysis does not give us a 360° view of the implications of assumptions made in relation to each aspect of the concept of health security.

### 3.1 Security for Whom?

Mainstream theories of International Relations (IR) and security studies assume the state as the main referent object of security.^[^ ^65^ ^]^ Current health security analysis is also predominantly premised on this assumption. While some studies assert it explicitly,^[^ ^39^, ^66^, ^67^ ^]^ most do so implicitly by either discussing security only in relation to health threats that challenge states’ strategic interests,^[^ ^29^, ^68^, ^69^ ^]^ or by examining health as a foreign policy or national security concern, both of which are by definition state‐centric.^[^ ^28^, ^38^, ^40^, ^70^ ^]^ The main consequence of focusing on the state as the sole referent object of security is that only a narrow set of health problems, which are perceived to cause acute state instability, state failure or destabilize other interstate relations, qualify as relevant security challenges, while many others remain ignored, excluded, and understudied.

Direct threats to state security are perceived to emanate from diseases that cause large‐scale morbidity and mortality, cross national borders and affect populations, rather than just individuals.^[^ ^71^ ^]^ It has been argued that such diseases are destabilizing for states only in extreme circumstances—by affecting the health status of military personnel or peacekeepers,^[^ ^69^ ^]^ by undermining state structures and political stability, by exacerbating existing political instability, or by impacting the labor force and the economy, and reversing years of economic development.^[^ ^72^, ^73^, ^74^ ^]^ Even in these situations, however, the impact of ill‐health would be most acutely felt by individuals and communities. Diseases (both communicable and noncommunicable) pose an existential threat to individuals, affecting their own and their families’ sense of daily security, stability, predictability, well‐being, economic, and development prospects, in a way that cannot be experienced at state‐level. What is more, ill‐health is the most relevant existential threat to people with 9 out of the top 10 causes of death worldwide being health‐related,^[^ ^75^, ^76^ ^]^ which makes a strong argument for promoting human‐centered security. Baldwin argues that conceptually, and for purposes of specifying the concept of security, individuals, states, the international community can all be considered relevant referent objects of security.^[^ ^20^ ^]^ But while such analysis is central to gaining a more accurate and nuanced understanding of dynamic and evolving security problematics globally, existing security, and international relations paradigms are poorly equipped for such multiscalar analysis.

### 3.2 Security for Which Values?

Traditional IR theories consider the preservation of the sovereignty, autonomy, and territorial integrity of states as core values to be secured.^[^ ^77^, ^78^, ^79^ ^]^ The raison d’etre of the state is to protect itself from external invasion or transgression, they argue, and it is only by ensuring its own security that the state is able to guarantee the security of its people. Baldwin argues that in practice, other values are sometimes added to the national security agenda and that the values, which are being pursued by security politics ought to be clearly specified, so as to assist analysts in evaluating their relative importance and resource needs in comparison with other policy objectives.^[^ ^20^ ^]^ Importantly, Baldwin further argues against specifying security objectives in absolute terms, because absolute security is unattainable, which justifies his next question about the degree of security sought in a particular issue area.

Political and normative developments in international politics demonstrate that the spectrum of values that states have agreed to secure is growing. This is illustrated by the emergence of concepts such as human development,^[^ ^80^ ^]^ human security,^[^ ^81^ ^]^ responsibility to protect.^[^ ^82^, ^83^, ^84^ ^]^ Increasing attention has been paid to the protection of civilians in inter‐state conflicts through the growing body of international humanitarian law. States have further committed to seeking individual criminal responsibility for acts of genocide, war crimes and crimes against humanity by accepting the jurisdiction of the International Criminal Court and thus offering further protection for people from the exigencies of uncontrolled power.^[^ ^83^ ^]^ The influence that these norms and values are having on security policy is understudied by both traditional and critical security studies. These norms demonstrate a shift in values toward securing and protecting individual life and population well‐being, alongside traditional state security.

Most analyses of health security do not engage in depth either with this evolving international normative context or with security studies paradigms that are more human‐centered. Health security studies struggle to effectively reconcile the values pursued by public health—i.e., the protection and promotion of the health of communities and traditional security studies—identified as existential threats to states.^[^ ^16^ ^]^ The values to be secured according to this literature, therefore, continue to be the stability and integrity of states—by means of preventing internal instability and state vulnerability that may be caused by high morbidity and mortality, and external instability caused by state failure and conflict. Since assumptions about what values ought to be secured are implicit in the health security literature, the implications of these choices for security policy have not been sufficiently evaluated.

### 3.3 How Much Security?

At first glance, this question might appear futile and its answer obvious—surely, more security is always better. Baldwin clarifies its significance—“[i]n a world in which scarce resources must be allocated among competing objectives, none of which is completely attainable, one cannot escape from the question ‘How much is enough?” and one should not try.”^[^ ^20^ ^]^ Morgenthau sets out the realist position on this question: “all nations must allocate their scarce resources as rationally as possible” to guarantee national survival.^[^ ^85^ ^]^ Offensive and defensive neorealists agree, but disagree on whether state security is best achieved through gaining the “appropriate amount of power”^[^ ^78^ ^]^ or through the maximization of power relative to other states.^[^ ^86^ ^]^ In practice, increasing spending on the pursuit of some values invariably means reducing spending on the pursuit of others. In a world where most states are not “great powers,” the scope of security politics is richer and much more nuanced than presented by IR theories.

The question of “how much security” has not been addressed at great length in the health security literature. The literature has broadly operated on the assumption that should health‐related issues be “securitized” successfully, they will get the resources needed, which is in line with traditional security thinking on the issue. The scale of resources committed to addressing health crises, however, has so far been decided on an ad hoc basis, primarily by donors (public or private), and has often reflected the perceived proximity or scale of a health threat to national security as argued by Rushton.^[^ ^64^ ^]^ Thus, for example, a recent round of replenishment for the Global Fund to fight HIV/AIDS, Tuberculosis and Malaria saw donors pledge nearly $13 billion.^[^ ^87^ ^]^ These diseases carry a similar burden of morbidity and mortality as some noncommunicable diseases, which have not received nowhere near as much funding.^[^ ^10^, ^88^, ^89^, ^90^, ^91^, ^92^ ^]^ Nuclear defense spending in the UK and US, for example, far outweighs pandemic‐preparedness spending, as highlighted by the current coronavirus pandemic. Further analysis of the theory and practice of framing health security challenges is therefore urgently needed, with attention drawn specifically to existential threats facing individuals and communities.

### 3.4 From What Threats?

Threats to security are traditionally defined as being external to the state, predetermined by the anarchic structure of the international system, and military in nature.^[^ ^78^ ^]^ The sharp decline in violent interstate conflicts and conflict‐related deaths, however, as noted by the Human Security Report Project,^[^ ^93^ ^]^ threatens to deprive these studies of an object. Does this mean, then that states and people are secure? One could hardly say so. With the majority of conflicts taking place within states and involving at least one nonstate armed group,^[^ ^94^, ^95^ ^]^ (with the intensification of violence against civilians, increasing intractability of conflicts and the spread of violent conflict to middle‐income countries (Iraq, Syria, Ukraine), assumptions about the nature and causes of conflict are continuously being challenged.^[^ ^95^ ^]^ The human cost of these conflicts is currently born extensively by civilians.^[^ ^96^ ^]^ In addition to conflict, people across the world lead daily battles for survival against disease, poverty, malnutrition, environmental degradation, climate change, lack of access to clean water, safe food, basic health services, against political oppression, gender‐based violence, and so on. In this context, Baldwin’s argument that there is no reason to limit the concept of security to narrow, vague references at the expense of referring to practical threats that are comported with common usage,^[^ ^20^ ^]^ is particularly relevant.

The health security literature has broadly kept in line with traditional security approaches on this question as well, by focusing analysis primarily on issues with a crossborder impact on national security, which has led to an overall narrow focus on health‐related causes of insecurity—namely, emerging and re‐emerging infectious diseases (ERIDs) and bioterrorism.^[^ ^21^, ^64^, ^97^, ^98^ ^]^ Some scholars have acknowledged that this focus is too narrow, proposing the inclusion of other issues such as internal state instability and illicit activities and an increased engagement of health security with public health and not just with the concerns raised by the foreign policy and security communities.^[^ ^66^ ^]^ This, however, is only a marginal broadening of the agenda, which fails to engage with two central questions—the protection of individuals and communities from danger, hazard and risk; and the much more complex question of whether security policy is just about negative security “security from” or whether consideration should be given to positive security as “security to.”^[^ ^99^ ^]^ McSweeney also talks about the importance of considering “structural” threats, namely, the unintended consequences of social action^[^ ^99^ ^]^ – the structure of the global economy, the pattern of power relations and dependencies within it, the profound influence of the food, tobacco and alcohol industry on government policy, gender inequality, levels of relative and absolute poverty, income inequality, etc. This is not to say that the health security literature is not cognizant of these, just that they have not been explored systematically and in sufficient depth, because too much attention has often been focused on dealing with acute threats.

### 3.5 By What Means?

Sovereignty grants states legitimate monopoly over the use of violence. Employing the sovereign authority of states to respond to security problems is usually synonymous with the threat or use of military force or other types of coercive action. Baldwin argues that the “specification of this dimension of security is especially important in discussions of international politics” and expresses concern that tendencies to define the field entirely in terms of the threat and use of military force “can prejudice discussion to favor of military solutions to security problems.”^[^ ^20^ ^]^ Improving and securing health, for example cannot be achieved by military means, even though military personnel and logistics can and have been utilized in emergency responses. Pursuing security through nonmilitary means requires a human‐centered focus and cooperative approach, where states engage not only with each other but also with a broad spectrum of nonstate actors. In security politics the state is increasingly becoming one actor among many, but with a key facilitating function in delivering security to individuals and communities.

Responses to health security challenges involve a broad spectrum of public and private actors, including intergovernmental organizations, inter‐agency cooperation, civil society organizations, philanthropic foundations, corporate actors, etc.—making for a complex governance architecture and a dynamic combination of various means and resources. The role of this panoply of actors has been explored in the context of global health governance,^[^ ^100^, ^101^, ^102^ ^]^ but not sufficiently so in the context of security politics, where the dynamics of governance interactions are distorted. In addition, promoting and improving health requires investment in infrastructure, education, knowledge development, in lifting people out of poverty, enhancing food and environmental security, all of which require concerted, cooperative efforts. This is a different model of thinking about security politics and appropriate security policy, compared with the zero‐sum game, military, confrontational approaches proposed by mainstream security studies. Some analysts promote the concept of “cooperative security,”^[^ ^60^, ^103^, ^104^, ^105^ ^]^ premised on the changing nature of security threats as well as changing practices of security governance. This is an emerging fields of security analysis on which health security studies ought to draw more extensively.

### 3.6 At What Cost?

Even though the assumption that security ought to be pursued at any cost is at the heart of traditional thinking about security politics, such conceptualization is unrealistic in most situations. As Baldwin points out—“costs always matter.”^[^ ^20^ ^]^ There are virtually no instances in practice where no restrictions on the means and costs of responding to a threat to security are in place, nor where other values are not competing for or having to be sacrificed in the distribution of scarce resources. This is not a question that has been examined in great detail either in mainstream security studies or in the health security literature, suggesting that analysts have adopted the traditional, exceptionalist frame of thinking about security, whereby a successful “securitizing move,” automatically guarantees the availability of “sufficient” funds and resources to address security threats.

Addressing threats to security stemming from ill‐health requires resources that go beyond the cost of medicines, as has become apparent through campaigns dealing with the spread of HIV/AIDS, tuberculosis, malaria, the fight against polio, and recent infectious disease crises—Ebola, Zika, and the Covid‐19 pandemic. The Global Health Security Agenda launched in 2014, by the United States together with 28 other states, WHO, the Food and Agricultural Organisation and the World Organisation for Animal Health, is one such attempt to promote activities aimed at strengthening “core capacities… of public health systems needed to protect global health security.”^[^ ^106^ ^]^ Analysis of the relative cost of security through the improvement of health and the alleviation of existential threats through the strengthening of health systems can be of particular importance in setting out domestic and global security priorities, but also in raising the required funds. Cost is in many situations an inhibiting factor in pursuing particular interventions, despite evidence of the need for the latter. It should therefore always be a significant consideration in any health security policy analysis.

### 3.7 In What Time Period?

Mainstream international relations theories do not make a significant distinction between long‐, medium‐, and short‐term security goals. Their atemporal approach to security is premised on the assumption that the causes of conflict and insecurity do not change over time, due to the unchanging character of the anarchic international system.^[^ ^107^ ^]^ Ahistoric realist and neorealist analysis seeks to justify the perpetual need to invest in military resources. In his brief discussion of this aspect of security, Baldwin warns that short‐term security politics often respond to an immediate threat, but a longer‐term strategy for security may well conflict with the short‐term approach.^[^ ^20^ ^]^

The existing health security literature does not tend to explore medium and long‐term policy horizons, despite the pertinence of such temporal considerations to a broader view of health security. The health security literature has mainly taken an interest in current crisis^[^ ^1^, ^3^, ^108^, ^109^, ^110^ ^]^ with the aim of understanding the politics and institutions involved in the responses to these. And while such analysis is important and relevant, conclusions often point toward the need for a medium and long‐term planning and investment. Improvements in overall health security require much more than pandemic preparedness measures, including investment in the development of healthcare infrastructure, health systems strengthening, training of medical personnel. Addressing the root causes of noncommunicable diseases, for example, may not be possible in the short‐term, as they necessitate regulation and preventive action, which takes time to negotiate and implement as well as longer term planning and infrastructure investment. Thinking about health security in differentiated time frames, therefore, could allow for a broader range of goals to be pursued, for more effective distribution of resources between acute and long‐term needs and for pursuing goals of prevention, while also providing care where needed.

## 4 Dimensions of Health Security

Baldwin’s concept of security provides a structured and comprehensive framework for thinking about health security. It promotes systematic thinking about the assumptions and practice of health security politics that is not confined by the rigid ontologies of traditional security paradigms, but is open, flexible, and practice oriented. This conceptual framework enables the combination of rich empirical insight from existing studies of health security with a broader spectrum of approaches to security studies, to not only enhance understanding of political dynamics, but to also generate pragmatic policy options, accommodating of normative considerations. This section sets out new parameters for health security analysis that go beyond the constraints of traditional security studies. These require further analysis of practice and engagement with alternative security frameworks to inform health security policy on how best to address persistent criticisms and shortcomings. Baldwin’s guiding questions are grouped in three categories—ontological, normative and material considerations, and discussed in turn.

### 4.1 Ontological Considerations—Security for Whom and by Whom?

Concerns about the security of individuals have continuously been embodied in new international legal norms and made part of the global policy agenda over the course of the last few decades. The post‐Cold War years “exposed the fragility of the state in the face of complex forces within it and of trans‐state limitations on its practical sovereignty outside it.”^[^ ^99^ ^]^ Instances of conflict, civil strife, political instability, state fragility, and now of the COVID‐19 pandemic are reminders that states are not always able or willing to guarantee the security of their citizens. The dominant view of security as a state‐centric concept has been presented by its proponents “not as an option, a choice, but as the only one which is valid and relevant… [but] the assumption of security studies which ignores the human dimension is contradicted by the practical dependence of policy‐makers and theorists alike on the human individual as the ultimate referent, or subject of security,” argues McSweeney.^[^ ^99^ ^]^

The argument in favor of foregrounding the security of individuals and communities in conceptual and theoretical debates is supported by practice. Its relevance is particularly obvious in the context of (ill)health, which is probably one of the most prominent existential threats to humans, alongside environmental and food security. If the survival of individuals is not safeguarded, the survival of social structures and institutions loses its significance. Contextualizing the security of individuals and groups in relation to and within state security is an area of security analysis that needs further attention in a changing landscape of political conflict—examples include the health security of populations in the context of civil war, failed or fragile states,^[^ ^111^, ^112^, ^113^ ^]^ or the provision of health‐services in territories held by nonstate groups, e.g., rebels, guerrilla groups, ISIS; or the security of women and girl refugees fleeing conflict.^[^ ^114^, ^115^ ^]^ Empirical evidence needs to be brought to bear on understandings of security politics in general and health security in particular.

In addition to analyzing the relationship between individual, group, and state security, attention needs to further focus on the “providers” of security, which increasingly include specialized nongovernmental organizations, public–private partnerships, philanthropic foundations, multilateral agencies, and others. It has been assumed that this dynamic governance architecture is still under the control of sovereign governments, but there is little evidence to support that, particularly in contexts of conflict, fragile or failing states such as Syria, Afghanistan, Yemen, South Sudan. Rushton and Williams’ “Partnerships and Foundations in Global Health Governance,”^[^ ^101^ ^]^ Harman’s “Global Health Governance,” and^[^ ^102^ ^]^ Jeremy Youde’s “Private Actors, Global Health and Learning the Lessons of History”^[^ ^116^ ^]^ are useful starting points in outlining the architecture of health governance, but further analysis is needed to reflect on the idiosyncrasies of security‐focused governance and politics. Gjorv (2012) advocates the need to adopt a multiactor security model to explore the patterns of security‐related governance, which she argues is prompted not only by normative considerations, but is a reflection of the empirical realities facing security practitioners, illustrating her argument with two examples civil‐military operations and climate change in the Arctic.^[^ ^62^ ^]^

Baldwin’s conceptualization of security demonstrates that restrictions on the referent object of security are superficial. When health‐related risks and challenges pose an existential danger, they need to be considered as security risks, in recognition that individual and community security is as relevant a consideration to state security and vice versa. Health‐related existential threats to individuals and communities are further exacerbated by poverty, political instability, state fragility, conflict, and civil strife. But since state security can both determine and be determined by the security of individuals and communities, and since there are other actors involved who impact or are impacted by such insecurities, a more comprehensive understanding of the politics and frameworks of health security policy making is urgently needed.

### 4.2 Normative Considerations—Security for Which Values? From What Threats? How Much Security?

Despite traditional theories of international relations discounting normative considerations in matters of security and national security, such considerations are always present. As discussed earlier, adopting a narrow state‐centric, militaristic view of security is both an option and a normative choice, and not the only possible or valid one. This is the premise of much critique from critical security studies, as illustrated by works such as Krause and Williams,^[^ ^117^ ^]^ Barkawi and Laffey,^[^ ^55^ ^]^ Booth,^[^ ^49^ ^]^ Peoples and Vaughan‐Williams,^[^ ^118^ ^]^ Sjoberg,^[^ ^119^ ^]^ Wibben,^[^ ^120^ ^]^ and Shepherd.^[^ ^121^ ^]^ Some analysts are further advocating consideration of security in terms of both positive and negative security, where negative security aligns more with traditional notions of security as “security from,” while positive security is seen as enabling and emancipatory—“security to.”^[^ ^62^, ^99^, ^122^ ^]^ Such a lens enables values such as human life, life in good health, life with dignity, to be placed at the center of security strategy and policy, which in turn demands that security politics become more inclusive, more protective, less focused on privileged views and experiences of security, more human‐centric. Framing security as a positive value creates space for considerations such as health system strengthening, the provision of primary care and universal health coverage, the prevention of noncommunicable diseases, to be given greater policy priority, which as analysts have argued would not only improve health outcomes overall, but could also strengthen health responses to acute crises.

Health is an important value on a global scale, as evidenced by the Constitution of the WHO (1948), the Alma Ata Declaration (1978), the International Health Regulations (2005), the Sustainable Development Goals (2015), along with the intrinsic value of human life, which is the bedrock of all international human rights norms, treaties, and declarations. If life and good health are the values to be secured, however, state policies would have to go beyond seeking to protect individuals and populations from emerging and re‐emerging infectious diseases and bioterrorism and take into account a broader spectrum of health‐related existential threats to people. Diseases posing significant risks to people in low‐ and middle‐income countries include among others neglected tropical diseases (NTDs)^[^ ^123^, ^124^, ^125^ ^]^ and noncommunicable diseases. NTDs’ burden of disease measured in DALYs ranked these diseases fourth after lower respiratory infections, HIV/AIDS, and diarrheal diseases, preceding malaria, TB, and measles.^[^ ^123^ ^]^ “Noncommunicable diseases (NCDs) are the leading cause of death globally and one of the major challenges of the 21st century.”^[^ ^126^ ^]^ An estimated 71% of all deaths globally in 2016 resulted from NCDs, the World Health Organisation (WHO) reports. Over the next 20 years, NCDs will cost more than USD 30 trillion, pushing millions of people below the poverty line.^[^ ^127^ ^]^ Much like other global problems, health insecurity disproportionately affects low‐ and middle‐income countries, as well as the poorest and often most disadvantaged strata of societies in high‐income countries. An infectious disease pandemic like COVID‐19 further worsens health outcomes by compacting morbidity, exponentially increasing mortality and creating a perfect storm even for the relatively well‐resourced health systems in high income countries.

Securing health and well‐being is an important goal in a dynamic portfolio of values that need to be protected. How much attention should be devoted to health overall, and to specific health concerns, or the needs of particular groups within this portfolio, are questions that needs to be examined further and in greater detail, drawing on studies of public health in individual states and across borders. The answer to the question “how much security” is also likely to vary over time. Analysis of the relative threat posed by a given health issue to individual, community, and state security is a valid consideration for health security politics—using a structured framework to enable comparative analysis is central to health security analysis. Due to the relatively high morbidity and mortality, the COVID‐19 pandemic has demonstrated that health threats can be elevated to almost absolute, primary status. Actions taken to contain the pandemic have included social distancing measures, limiting travel, shutting down economies, governments promising to pay salaries, support private businesses, etc., which are measures that appear unthinkable in most other cases. In the midst of this crisis, however, it is important to remember that pandemics of such scale and scope are relatively rare and to use COVID‐19 more as an extreme example than a baseline one.

### 4.3 Material Considerations—By What Means? At What Cost? In What Time Period?

Contrary to traditional security approaches premised on the use of military means, health security (whether broadly or narrowly defined) requires the employment of nonviolent, cooperative measures—including investment, humanitarian aid, development assistance, multiactor cooperation, coordination, sharing of information and expertise, etc. As discussed previously, the cost of addressing health security problems is significant, due to the need to establish and support a functioning health system, to train and retain professional staff, to create infrastructure that facilitates the functioning of the health system, but the cost of inaction is high and puts lives at risk. The challenges posed by public health emergencies of international concern and pandemics can be exceptionally far‐reaching and damaging—globally, locally, trans‐locally, as illustrated by the current spread of the SARS CoV2 virus. The human cost of this pandemic has been unprecedented in recent history, the economic costs are yet to be calculated with more than a quarter of the global population in lockdown, international travel restricted and economies shrinking fast. In a world of scarce resources, the means for securing health and the cost of doing it are pertinent policy considerations, which need to be examined in conjunction with the opportunity cost of both not investing in health security and of investing in a different field.

The short‐termism and immediacy of conventional security politics is counter‐productive in approaching problems such as anti‐microbial resistance, noncommunicable diseases, maternal and infant mortality. Even responses to public health emergencies of international concern, in the form of communicable disease outbreaks, have demonstrated the need to develop a systematic approach—including properly resourcing the work of the WHO, investing in health systems strengthening and infrastructure. The 2011 report of an Independent Review committee on the H1N1 response noted that “The world is ill‐prepared to respond… to a global, sustained, and threatening public health emergency” as health capacities were not on a path to timely, worldwide implementation.^[^ ^43^ ^]^ The international community collectively and states individually appear to have squandered the time since 2009 to prepare for the next global pandemic. The health systems in high‐income countries are buckling under the weight of the COVID19 pandemic. Concerns are growing over its effects the pandemic will have on low‐ and middle‐income countries. In the conclusion to their discussion of the global response to Zika virus, Gostin and Hodge point out that the apathy and short‐sightedness of the international community must change, as the consequences of fast‐moving epidemics are comparable with humanitarian crises, climate change, and war.^[^ ^6^ ^]^ Such analysis and current events clearly illustrate that planning has to include the short‐, medium‐, and long‐term and might be more effectively organized at the global level, as states are better off responding together than individually.

To sum up, this discussion of the dimensions of health security demonstrates that health security can be conceived of as focusing on the security of people, communities, and states, if we accept that health security politics are centered on the protection of the core values of life and life in good health. Since health security is concerned with issues that both pose an existential threat to people and also threaten and destabilize communities, its significance ought to be ranked relatively high. Health security politics need to be viewed both as being embedded within the existing normative context of human rights and as themselves promoting a range of values—including dignity, respect, nondiscrimination, emancipation, and empowerment. The pursuit of health security requires material resources like any other type of security. Part of the politics surrounding health challenges center on competition for attention and scarce resources. The resources required for the enhancement of health security can be significant, as they involve developing infrastructure, training health professionals, the delivery of care, ensuring the accessibility of medicines, disease prevention, health promotion, and strengthening health systems.^[^ ^128^ ^]^ Conceptualizing health security in this way, calls for a more holistic approach to encompass both the important work done through responses to global health emergencies and the need for medium‐ and long‐term policies, because in health, just like in strategic politics, prevention is always better than cure.

Thinking about health security in a systematic way simultaneously highlights the idiosyncrasy of the health security field compared with other fields of security politics and demonstrates the interconnectedness and overlaps between them. Baldwin’s concept of security provides a guiding framework, a structured conceptualization through which to rethink the way in which health security has been imagined. The flexibility of ontological assumptions that it provides opens possibilities for health security studies to connect with contemporary security paradigms, defying the stereotypes, and constraints of traditional thinking about security. At the very least, it provides a structured framework that allows for comparative analysis of competing accounts of security politics with diverse paradigmatic assumptions. The framework is able to accommodate not just conceptual debate but observations of and reflections on practice.

## 5 Conclusion

This article sought to contribute to debates about conceptualizing health security and understanding health security politics. It set out to challenge the use of traditional security paradigms, which obscure the significance of public health threats to individual and community security and well‐being. The current COVID‐19 pandemic has brought these issues to the fore with a much sharper focus than previous public health emergencies of international concern. The brief overview of the different denominations of health security demonstrated that the grounding of existing analysis in securitization theory and constructivist thought has been driven in part by the desire to validate the claim that health‐related challenges were indeed relevant security concerns, and in part by the need to fit within existing debates. Overall, as has been demonstrated, health security analysis has remained predominantly anchored to the securitization approach, despite critiques levied at the Copenhagen school by critical and feminist scholars.^[^ ^129^, ^130^, ^131^ ^]^ Health security analysis has only marginally engaged with related bodies of work in the fields of foreign policy, human security or with alternative security paradigms, which has limited the field’s dynamism, critical edge, and ability to influence policy debates.

This article applied Baldwin’s framework of the concept of security to existing conceptual and empirical studies of health security to demonstrate how narrowly health security has been conceptualized and how much more analysis is needed for a better understanding of this complex field. Traditional security analysis is broadly inhospitable to claims that health issues are a relevant security consideration, leading some analysts to reject the relevance of health to strategic security instead of questioning whether the way that security is framed and defined is still relevant to political and strategic realities and practice.

Baldwin’s framework helps liberate health security analysis from the dogmatic assumptions of traditional security theories, while at the same time providing a structure for rigorous, comprehensive and comparable conceptual debate. Experimenting with novel thinking about the ontological, normative and material considerations in health security can help push the boundaries not only of the health security field, but of security studies overall. Particular questions for further research emerge—e.g., about the relationship between individual, community and state security, about the way in which resources are allocated to specific fields of security politics, about the differences in short‐, medium‐, and long‐term planning in health security politics, about ways in which to evaluate the relative importance of competing security challenges, the relationship between perceptions and indicators, and so on. The exploration of these questions, based on a clear, explicitly defined concept of health security will promote more systematic thinking about security politics that is open, flexible, and practice oriented.

Taking a broader, more holistic and historically grounded approach to understanding the politics of health and security brings its own set of challenges. A comprehensive rather than parsimonious way of thinking would inevitably complicate analysis, as it incorporates more variables and tries to capture more, not less of the political and social complexities of security policymaking. Questions about normativity and ethics need to be considered. Drawing on knowledge across disciplinary borders is rarely unproblematic, as ontological, epistemological, and methodological differences may hinder multidisciplinary dialog. None of these difficulties, however, are insurmountable, as demonstrated by novel approaches to security studies, on which health security analysis ought to build.

The pressing needs to rethink the dimensions of health security has regretfully been validated by the unfolding COVID‐19 pandemic. Writing in the midst of this crisis, it is difficult to assess what the implications of this pandemic would be for societies, economies, health systems. The issues that the pandemic is bringing to the fore, however, are not new. Academics have grappled with and tried to draw attention to some of these for at least the last 20 years.^[^ ^132^ ^]^ SARS CoV2, the virus that causes COVID‐19, is a health security threat—make no mistake about it. This virus poses an existential risk to humans—it threatens individuals, but impacts on communities and on almost every aspect of societal life—family life, social relations and activities, culture, education, the economy, government. Governments around the world are using an unprecedented spectrum of measures to reduce morbidity and mortality, previously unseen in peacetime.

The WHO has repeatedly noted that the response to the pandemic would be most effective if states work together in a spirit of cooperation, solidarity, and care. States need people, businesses (including private health care providers) and voluntary organizations to support the pandemic response, which is an illustration of multiactor security politics coordinated by governments and intergovernmental organizations. There is no doubt that this pandemic will bring about change—the extent and nature of the change is currently unknown. What the pandemic has illustrated so far, however, is the need for health systems strengthening, for deepening of global coordination and cooperation, and a stark need to critically reflect on the way we conceptualize security in general and health security in particular without leaving the individual and communities out.

## Conflict of Interest

The author declares no conflict of interest.
